# A Quality Improvement of the Identification of Obesity in Patients With Mental Health Morbidity and Referral to Weight Management Services

**DOI:** 10.1192/bjo.2022.328

**Published:** 2022-06-20

**Authors:** Anjali Patel, Reshma Rasheed, Yathorshan Shanthakumaran

**Affiliations:** 1 New Vision University, Tbilisi, Georgia; 2 Rigg Milner Medical Centre, East Tilbury, United Kingdom

## Abstract

**Aims:**

A quality improvement project was undertaken to counteract obesity in patients with mental health morbidity. The exponential trend of increased antidepressant prescribing (SSRI, SNRI and anti-psychotic medication) has created a trend towards weight gain in patients. An audit of the Serious Mental Illness (SMI) register and depression registers was conducted in a population of 591 patients. Those patients identified as obese were offered referral to the local authority weight management services.

**Methods:**

Patients have a body weight and BMI calculation with their twice yearly mental health and medication review and those whose BMI met the obesity criteria were offered referral to the local authority for 12 weeks weight management services.

**Results:**

Of the SMI and depression register 189 (32%) patients met the criteria for referral to the weight management program. Of these 154 (81%) patients accepted the local NHS weight management program, 35 (18%) of patients declined the NHS weight management program.

**Conclusion:**

Weight gain is a known side effect of antidepressant medication SSRI and SNRIs and Anti psychotic medication resulting in increased risk of obesity and cardiovascular and metabolic disease. The QI program was undertaken to counteract these changes with referral to weight management services to address the weight gain the patients were experiencing.

This quality improvement service was done to help patients across three surgeries lose weight in an effective and educational manner. We found a high rate of acceptability of referral to weight management services when offered as patients themselves were aware of the weight gain. A review of positive changes in the BMI after referral to the weight management program will be undertaken at 6 and 12 months to evaluate its acceptability and effectiveness. We advocate sensitive counselling of the risks of weight gain and regular monitoring of body weight throughout the span of the prescribing of these weight gaining agents.

